# Promoting Sustainable and Healthy Diets to Mitigate Food Insecurity Amidst Economic and Health Crises in Lebanon

**DOI:** 10.3389/fnut.2021.697225

**Published:** 2021-06-25

**Authors:** Nahla Hwalla, Lamis Jomaa, Fatima Hachem, Samer Kharroubi, Rena Hamadeh, Lara Nasreddine, Farah Naja

**Affiliations:** ^1^Nutrition and Food Sciences Department, Faculty of Agriculture and Food Sciences, American University of Beirut, Beirut, Lebanon; ^2^Nutrition and Food Systems Division, Food and Agricultural Organization of the United Nations, Rome, Italy; ^3^Department of Clinical Nutrition and Dietetics, College of Health Sciences, Research Institute of Medical & Health Sciences, University of Sharjah, Sharjah, United Arab Emirates; ^4^Faculty of Agriculture and Food Sciences, American University of Beirut, Beirut, Lebanon

**Keywords:** sustainable diet, healthy diet, food consumption, food security, quadratic optimization, Lebanon

## Abstract

**Introduction:** Lebanon, a middle-income Eastern Mediterranean country, continues to face detrimental economic, health and socio-political challenges that are further exacerbated by the COVID-19 pandemic. In parallel, the country has been experiencing a remarkable nutrition transition that has contributed to the burden of malnutrition and non-communicable diseases, all imposing serious repercussions on people's livelihoods, food security, and health. Such circumstances have prodded public demand for guidance on affordable, healthy, and sustainable dietary choices to alleviate the burden to this emerging unfortunate situation.

**Objective:** The purpose of this study is to provide evidence-based sustainable and healthy dietary recommendations which balance the tradeoffs among the health, environmental footprint and cost dimensions of sustainability, while closely resembling the usual food consumption pattern.

**Methodology:** Data from the latest available national food consumption survey was used as the usual food consumption pattern of Lebanese adults. Optimized dietary patterns were calculated using the optimization model Optimeal which produced patterns most similar to the usual diet and simultaneously satisfying the three main sets of constraints: health, environmental footprints, and cost. The identified healthy and sustainable dietary options were vetted by multiple key stakeholders from the government, academia, international, and national non-governmental organizations.

**Results:** Compared to the usual intake, the optimized diet included higher intakes of whole grain bread, dark green vegetables, dairy products, and legumes, and lower intakes of refined bread, meat, poultry, added sugars, saturated fat, as compared to usual national mean consumption. The optimized dietary model resulted in a decrease in the associated environmental footprints: water use (−6%); and GHG (−22%) with no change in energy use. The cost of the optimized diet was not different from that of the usual intake.

**Conclusion:** An evidence-based sustainable and healthy diet was developed for Lebanon providing the population and policy makers with some answers to a complex situation. Findings highlight the need for the development of sustainable food based dietary guidelines for Lebanon to promote diets that are healthy, sustainable, culturally acceptable, and affordable and that can alleviate food insecurity among the general population.

## Introduction

Since October 2019, Lebanon has been facing dramatic economic, health, and socio-political challenges that have been further exacerbated by having one of the highest rates of COVID-19 worldwide (1). Civil unrest, sudden restriction on access to foreign currencies, a reduction in financial ratings, and the COVID-19 pandemic have raised concerns over the livelihood of the Lebanese population (2). In fact, the Ministry of Social Affairs estimates that the pandemic's effects on top of the economic crisis will lead to an increase by 50% in poverty and unemployment rates (3). As such, the combined impact of the COVID-19 pandemic and the economic freefall constitutes a unique and unprecedented situation, this country is facing, and imposes detrimental repercussions on people's livelihoods, food security, and health. This fast deterioration of economic conditions and COVID-19 pandemic in Lebanon, are affecting all pillars of food security including; availability, access, and utilization, which is now being further aggravated by movement restrictions, loss of income, and price inflation of food and non-food items (4). Given that the country relies heavily on food imports, the massive explosion that destroyed a significant part of the Port of Beirut on 4 August 2020 has worsened the food security situation (2). The United Nations estimates that over 50% of the Lebanese population might be at risk of failing to access basic food needs (2). This situation adds to an existing triple-burden of malnutrition in the country including hunger, micronutrient deficiencies, and an escalating burden of obesity and non-communicable diseases (NCDs), which all together negatively impact an already failing health system, leaving the population in extreme vulnerability (5–7). The burden of NCDs in Lebanon, including cardiovascular diseases, diabetes, cancer, and chronic respiratory diseases, remains the largest component of the country's health profile with 91% of all deaths attributed to NCDs (8). This is attributed in part to a gradual change in the national food consumption pattern, marked by an erosion of the Lebanese Mediterranean Diet and an increased predominance of the Western diets that are characterized by increased consumption of animal-derived food products that are high in energy, fat, added sugars, and salt, and a decreased intake of plant-based food, such as fruits and vegetables, ultimately leading to a decrease in the intake of dietary fibers and complex carbohydrates (9–12). In a context that operates with an already stressed food security system, further, vulnerabilities to food insecurity, malnutrition and obesity imposed by the COVID-19 pandemic and the economic freefall are expected to magnify disparities in healthy living behaviors, perpetuating a vicious synergy of complex yet preventable nutrition conditions that contribute to the exacerbation of diet-related NCDs and undernutrition (13). Such circumstances have prodded public demand for guidance on what would constitute affordable and healthy dietary choices and food consumption recommendations to alleviate the burden to this emerging unfortunate situation.

Thus, in light of the current and deteriorating food security situation, Lebanon is in dire need for guidance on culture-specific sustainable diet options that are healthy, affordable, and more sustainable (14–16). It is expected that proposing sustainable diet options that take into consideration the economic, health, and sustainable aspects of consumption may alleviate some of the health and food insecurity burden in the country and ease the concern of the population on what constitute adequate, affordable, and beneficial food choices.

Health, affordability and the environment are the three key components of food consumption which need to be balanced to provide a sustainable diet in line with health recommendations. Mathematical programming has long been used to identify adequately healthy, environmentally friendly, and affordable human diets (17). Results from an optimization model can be used to propose policy interventions to promote the consumption of healthy and environmentally sustainable foods (17). Therefore, the purpose of this study is to derive a healthy and sustainable diet, by defining healthy and sustainable food options, identifying changes to usual dietary practices, and suggesting feasible tradeoffs for Lebanese adults. This will be achieved by using an optimization mathematical model that generates optimal diet solutions that are healthy, of low cost and satisfy the adequate nutrient needs of adults in Lebanon taking into consideration environmental sustainability aspects. The results will provide policy makers and key stakeholders with the needed knowledge on how to mitigate the current precarious health and food insecurity situation by promoting a sustainable food consumption pattern that may be used to develop sustainable food based dietary guidelines (SFBDGs) for Lebanon that concomitantly address health, economic, and environmental sustainability dimensions.

## Materials and Methods

In this study, a dual approach that involves calculations and expert judgement were used. With regards to the calculations, a mathematical approach to compute an optimized dietary pattern for Lebanese adults, given a set of constraints and objective functions was used. Constraints were set for food items within food groups based on the World Health Organization (WHO), Mediterranean Diet (MD), and EAT-Lancet recommendations for sustainable and healthy consumption (18–20), supported by expert opinion. Calculations were made to resemble the usual food consumption pattern in Lebanon as closely as possible. Minimum and maximum constraints for nutrients and energy were selected based on DRIs' estimated average requirements and tolerable upper intake levels (21), and considerations with respect to environmental impact and cost were taken in accordance with the values of EFP and cost of the usual consumption pattern. The optimized dietary pattern was the pattern most similar to the usual diet (objective function). The results were used to identify and suggest food consumption changes, policy options that are sustainable, healthy, and emanate from the context of the current food situation in the country. The results and recommendations were consulted with and supported by various stakeholders, representatives from the government and the food industry and food and nutrition experts. Stakeholders meetings were conducted at different stages of the project, in order to build consensus on; (1) the constraints identified from the literature, (2) the results of the pilot test of the optimization, (3) the final results of the optimization, (4) the developed recommendations. The methodology used could be emulated in countries of the region with considerations to local socioeconomic and health situations to guide the development of sustainable diets and subsequently SFBDG.

### Study Population

The data for the Lebanese national consumption were derived from a national cross-sectional survey conducted in Lebanon during 2008/2009. Details about the protocols used in this survey is published elsewhere (22). The survey included a nationally representative sample of individuals, selected from households using a stratified cluster sampling, whereby, the Lebanese governorates constituted the various strata while districts within governorates were considered clusters. Within clusters, households were selected at random using probability proportional to size sampling and considering the distribution of the Lebanese population (by sex and 5-year age group) estimated by the Central Administration for Statistics in Lebanon (2004) (23). In each household, one adult and one child/adolescent were selected for participation, using a household roster. The field work was conducted between May 2008 and August 2009 with a final sample including 3636 subjects (refusal rate 10.7%). For the purpose of this study, data pertinent to adults (≥20 years old) participating in the 2008/2009 national survey, who had complete data, were included (*n* = 2177).

Dietary intake was collected from participants using the 24-HR multiple pass food recall (MPR) 5-step approach, developed by the United States Department of Agriculture (USDA) (24), and analyzed using Nutritionist Pro software (version 5.1.0, 2014, First Data Bank, Nutritionist Pro, Axxya Systems, San Bruno, CA, USA). For the current study, composite and mixed dishes were dissected into individual food items.

### Food Groups

Using data of the 24 h, intakes of various food groups were calculated. The food groups were selected to be in line with the WHO, MD, and the EAT-Lancet dietary guidelines. Individual food items were reviewed by independent nutrition experts to decide on their inclusion into the selected food groups. The food items which were consumed by Lebanese adults but their consumption is recommended to be substantially limited were excluded entirely. Such food items include; processed meat, sweets, salty snacks, sugar-sweetened beverages, and diet soda (*additional food groups*). The change in daily caloric intake after excluding the additional food groups was taken into consideration during the calculation of the usual dietary intake. An overview of the food groups and the food items within each food group, included or excluded from the optimization calculations, is described in Supplementary Table 1.

### Optimization Calculations

#### Health Constraints

The basic principle used to develop the sustainable and healthy diet was that recommended amounts of foods would deliver 100% of the essential nutrients. This could be achieved when 85% of the total energy was provided by food in the sustainable diet.

#### Micronutrients Constraints

To ensure the adequate intake of micronutrients among Lebanese adults, the Estimated Average Requirement (EAR) of each micronutrient, which is the average daily level of intake estimated to meet the requirements of 50% of healthy individuals, was used as a minimum constraint, and the Tolerable Upper Intake Level (UL), which is the maximum daily intake unlikely to cause adverse health effects, was used as the maximum constraint, on micronutrients to develop a healthy and sustainable diet that meets the nutritional needs of Lebanese adults (25–28) (Table 1).

**Table 1:** List of constraints for energy and nutrients used in the optimization calculations in the development of a sustainable and healthy diet.

**Nutrient or energy**	**Minimum**	**Reason for minimum**	**Maximum**	**Reason for maximum**
Energy	–	–	2,000 kcal^*^	Health
Macronutrient	Lower value of recommended range^†^	Health	Higher value of recommended range^†^	Health
Total fat	20% of energy		35% of energy	
Total protein	10% of energy		35% of energy	
Total carbohydrates	45% of energy		55% of energy	
Fatty acids with maximum intake	–	–	Recommended maximum Intake^‡^	Health
Saturated fat				
Trans fat				
Fiber	Recommended minimum intake^‡^	Health	–	–
Nutrients with EAR and UL	EAR^†^	Health	UL^†^	Health & food safety
Vitamin A				
Vitamin C				
Calcium				
Folate				
Zinc				
Iron				
Niacin				
Nutrients without UL	EAR^†^	Health	–	–
Thiamin				
Riboflavin				
Vitamin B12				

* *Based on 2013 Lebanese Food Based Dietary guidelines*.

† *IOM for adults, dietary reference intakes for energy, carbohydrate, fiber, fat, fatty acids, cholesterol, protein, and amino acids (2002)*.

‡ *World Health Organization Recommendations, World Health Organization (20). Healthy diet (No. WHO-EM/NUT/282/E). World Health Organization. Regional Office for the Eastern Mediterranean*.

*EAR, Estimated Average Requirements; UL, Upper Level*.

#### Macronutrients Constraints

The acceptable macronutrient distribution range was based on the recommendations of the Institute of Medicine (IOM) for adults (29). Fat, carbohydrate and protein intakes in grams/day were restricted to provide a maximum of 2,000 kcal per day as suggested by the 2013 Lebanese Food Based Dietary Guideline (30), to satisfy the caloric needs of Lebanese adults. As for the constraints on saturated fat, trans fat, and fiber intake, the recommendations indicated by the World Health Organization were used (20) (Table 1).

#### Food Groups and Food Items Constraints

Minimum and maximum constraints for daily intake of food were set to meet (1) the food groups recommendations of the WHO, MD, and EAT-Lancet guidelines and (2) the percent distribution of food items within each food group as consumed by the Lebanese population in the national consumption data (Table 2). The constraint for the percentage contributions of refined and whole grain bread to “Cereals and Grains” was calculated by summing the contributions of these two food items in the usual dietary intake and dividing the sum equally among refined and whole wheat bread. Furthermore, vegetable intake was not subjected to any constraint since, according to the 2013 Lebanese FBDG, Lebanese adults are recommended to consume a variety of vegetables without constraints (30).

**Table 2:** Food group/item minimum and maximum constraints.

**Food group**	**Reference diet (National consumption)**	**Percentage distribution from food group**	**Based on recommendations**	
			**Minimum g/day**	**Maximum g/day**
Cereals & grains	**233.59**		185.00^*^	300.00^*^
Bread (white/refined)	137.55	32%	59.50	96.50
Bread (whole grain)	12.81	32%	59.50	96.50
Cooked bulgur	6.83	3%	5.41	8.77
Cooked rice	56.70	24%	44.90	72.80
Ready to eat breakfast cereals	1.79	1%	1.42	2.30
Cooked pasta	17.92	8%	14.20	23.00
Fruits	**155.43**		160.00^*^	240.00^*^
Fresh fruits	135.71	87%	140.00	210.00
Dried fruits	1.04	1%	1.08	1.61
Fresh fruit juices	18.67	12%	19.20	28.20
Vegetables	**217.33**			
Dark green	47.26	22%	–	–
Red/orange	77.11	35%	–	–
Other vegetables	92.96	43%	–	–
Starchy vegetables	**44.08**		40.00^*^	80.00^*^
Potatoes	42.07	95%	38.20	76.30
Corn	2.01	5%	1.82	3.65
Dairy products	**105.93**		200.00^*^	400.00^*^
Milk	17.69	17%	33.40	66.80
Yogurt	33.15	31%	62.60	125.00
Cheese	35.82	34%	67.60	135.00
Labneh	19.28	18%	36.40	72.80
Meat	**54.82**	**–**	11.20^*^	22.40^*^
Poultry	**41.15**	**–**	23.20^*^	46.40^*^
Fish	**15.85**	**–**	22.40^*^	80.00^*^
Eggs	**9.71**	**–**	10.40^*^	20.00^*^
Legumes	**19.84**	**–**	**40.00**^‡^	**80.00**^‡^
Nuts & seeds	**13.56**	**–**	**–**	**60.00**^‡^
Added sugar	**8.38**	–	**0.00**	**24.80**^†^
Unsaturated added fat	**44.27**	–	**0.00**	**64.00**^‡^
Saturated oil	**2.44**	–	**0.00**	9.44^*^

* *EAT LANCET recommendations*.

† *World Health Organization recommendations*.

‡ *Mediterranean Diet recommendations*.

#### Environmental Footprints Constraints

In order to ensure that the derived healthy and sustainable diet does not use more environmental resources as compared to the usual food consumption, its environmental footprints were constrained to match those of the usual national consumption pattern (9) (Table 3).

**Table 3:** Environmental footprints and cost maximum constraints.

	**Unit**	Maximum constraint^*^
Water	L	1,370
Energy	MJ	17.5
GHG	kgCO_2_eq	1.91
Cost	L.L.	16,700
	$ (L.L.3,900/$)	4.28

* *These values exclude EFPs and Cost of additional food groups*.

* *The maximum was set to match the values of the reference diet*.

Water use, energy use, greenhouse gas (GHG) emissions, per 1 kg of each of the food items/groups included in the national consumption were calculated using a review of existing LCAs (Table 4). In order to select the LCAs utilized in the estimation of the footprints, priority was assigned to those conducted in Mediterranean or neighboring countries that possess a comparable climate to Lebanon (9). A detailed description of the LCAs that were included in the derivation of the EFPs metrics used in this study is available elsewhere (31).

**Table 4:** EFP (Water, Energy, and GHG) per 100 g for each food group used in the optimization.

**Food Groups**	**EFP/100 g**		
	**Water (L)**	**Energy (MJ)**	**GHG(CO2eq)**
Cereals and grains			
Bread	50.80	1.61	0.09
Whole grain bread	50.80	1.61	0.09
Cooked burgul	58.40	1.61	0.09
Cooked rice	133.00	1.91	0.26
Ready to eat breakfast cereals	48.40	1.61	0.09
Cooked pasta	52.10	1.78	0.13
All fruits			
Fresh fruits	63.30	0.53	0.04
Dried fruits	376.00	8.62	0.64
Fresh fruit juices	57.30	1.19	0.08
All vegetables			
Dark green vegetables	33.50	2.69	0.18
Red/orange vegetables	2.20	1.60	0.05
Other vegetables	18.80	2.42	0.06
All starchy vegetables			
Potatoes	24.90	0.05	0.01
Corn	55.70	0.27	0.04
Dairy products			
Milk	54.80	3.17	0.37
Yogurt	92.10	0.58	0.04
Cheese	439.00	0.58	0.04
Labneh	92.10	0.58	0.04
Protein rich foods			
Meat	814.00	3.78	1.62
Poultry	326.00	2.20	0.37
Fish	125.00	7.98	0.35
Eggs	271.00	1.10	0.35
Legumes	207.00	0.2	0.05
Nuts & seeds	494.00	0.50	0.04

#### Cost Constraints

In collaboration with the Lebanese Ministry of Economy and Trade (MoET), the cost of the usual mean national intake was calculated based on the market cost of food items consumed in the national intake during October 2020. The cost of each food groups was calculated based on the mean cost of the different food items constituting this group. For example, the cost of dried fruits was calculated by summing the average cost of 1 g of dried kiwi, mango, figs, strawberries, prunes, ginger, apricots, pineapple, and cranberries. These dried fruits are consumed by Lebanese adults as identified from the national consumption survey (2008/2009).

The cost of the usual mean national intake including all food items consumed by Lebanese adults, is L.L. 24,000 this is equivalent to $6.15 at a rate of L.L.3,900 per dollar. After excluding food items that are not recommended to be included in the diet (processed meat, sweets, salty snacks, added sugars, sugar-sweetened beverages and sodas), the cost of the mean national intake after the economic crisis in Lebanon is L.L. 16,700 which is equivalent to $4.28 at a rate of L.L. 3,900 per dollar. Cost constraints were capped to the cost of the usual Lebanese diet after excluding all food items that were not included in international recommendations (Table 3).

#### Optimization Model

The optimization calculations were performed using the optimization model in Optimeal software (32). Optimeal is a tool that uses optimization to solve dietary questions that involve sustainability as well as nutritional and cost parameters. The dietary pattern generated was a product of an optimization process that complied with all the set constraints and was most similar to the Lebanese usual diet (objective function) for reasons of cultural acceptability and feasibility. In the optimizations, the similarity of the two diets is defined as minimizing the sum of the squared deviations in food group quantity (grams) of the optimized diet and the consumption in the Lebanese National Food Consumption Surveys (2008/2009). This is known as quadratic programming. The method for quadratic programming can be formulated as follows (33).

$$\begin{array}{r} \text{Minimize }\frac{1}{2}{\text{x}}^{T}Q\text{x}+\;{\text{c}}^{T}\text{x}\\ \text{Subject to A}\text{x}>b \end{array}$$

where, for *m* nutrient constraints and *n* food items, the *m* by *n* matrix A consists of the nutrient contents for each food item and the vector **b** = (*b*_1_, …, *b*_*m*_) consists of the minimum nutrient requirements. The matrix *Q* along with vector **c** in the objective function consist of the cost per serving for each food item and **x** = (*x*_1_, …, *x*_*n*_) is the vector of the quantity of each food item. Note that **x**^T^ denotes the vector of transpose of **x**. The optimum was solved for using the quadratic programming algorithm in the newly developed Optimeal software. Optimeal software was used as a tool that utilizes quadratic programming to solve dietary questions that deal with many constraints such as sustainability, nutritional adequacy, and cost.

Note that ordinary linear programming is another mathematical technique that allows the generation of optimal solutions and is typically used in practice. However, the reasoning behind using the principle of quadratic programming here was to give preference to more food groups but less amount of changed grams per food group over a large change in one food group. For example, a preference for 10 g deviation in five food groups (sum of squared deviations = 5 × 10^2^ = 500), rather than a deviation of 5 × 10 = 50 g in one food group (squared deviation = 2,500). In addition, the result of quadratic programming can be more realistic when investigating future diets.

#### Vetting by Multiple Key Stakeholders

In order to build consensus on the process of optimization and the constraints used to develop a healthy and sustainable diet, stakeholders' meetings were conducted at different intervals of the study. The stakeholders represented various national and international entities, including the International Center for Advanced Mediterranean Agronomic Studies (CIHEAM), Food and Agriculture Organization (FAO), United Nations Economic and Social Commission for Western Asia (ESCWA), Lebanese Ministry of Economy and Trade (MOET), Lebanese Ministry of Agriculture, Fair Trade Lebanon, Syndicate of Lebanese Food Industries, and the Lebanese Ministry of Public Health. The results of the optimization calculations were studied by the expert panel in order to identify the most appropriate optimized solution. Expert opinion was first sought-after pilot testing the optimization, using all the constraints identified from international references. The results were disclosed with all stakeholders. The suggested recommendations and changes voiced by the different stakeholders were taken into consideration. A revised optimization was conducted to include all the recommended changes in the selected constraints along with the new parameters identified during the expert of panel meeting. A second stakeholders' meeting was held after the final optimization was performed in order to build consensus on the findings and help in the development of policy options for the adoption of the suggested healthy and sustainable diet. This was concluded in an iterative process according to the panel of experts' judgement taking into account the constraints and results of the optimization calculations.

## Results

### Results of Optimization Calculations

Table 5 presents the results of the optimization calculations in grams for the various food groups and items. The resulting model provided a dietary pattern that met all the constraints. While large variations between the usual intake and the optimized models were observed for certain foods groups, little variations were noted for others.

**Table 5:** Results of the optimization calculations, with the percent change from national consumption.

**Food groups**	**Optimized diet (quadratic) g**	**Percent change from national consumption**
		**%**
Cereals & grains		
Bread (white/refined)	59.5	(−57%)
Bread (whole grain)	89.6	(+599%)
Cooked bulgur	8.77	(+28%)
Cooked rice	44.9	(−21%)
Ready to eat breakfast cereals	1.42	(−21%)
Cooked pasta	14.2	(−21%)
Fruits	160.28	(+3%)
Vegetables	283.8800	(+31%)
Starchy vegetables	78.12	(+77%)
Dairy products	211.0000	(+131%)
Meat	11.2	(−80%)
Poultry	23.2	(−44%)
Fish	22.4	(+41%)
Eggs	10.4	(+7%)
Legumes	80	(+303%)
Nuts & seeds	15	(+11%)
Added sugar	3.61	(−57%)
Unsaturated added fat	0	(−100%)
Saturated oil	0	(−100%)

According to the results of the optimization, the optimized diet highlights the need for a significant increase in the consumption of; whole grain bread (+599%), dark green vegetables (+81%), dairy products (+131%), and legumes (+303%), and a significant decrease in the consumption of; refined grain bread (−57%), meat (−80%), poultry (−44%), added sugars (−57%), unsaturated added fat (−100%), and saturated fat (−100%), as compared to the national mean consumption.

These changes will result in a decrease in the associated EFPs: water use (−6%); and GHG (−22%) with no change in energy use. The cost of the optimized diet was not different from that of the usual intake (L.L.16,700). Furthermore, this optimized diet will result in an increase in the diet's composition of carbohydrates (+18%), protein (+22%), and fiber (+80%), and a decrease in total fat (−33%), saturated fat (−7%), and trans-fat (−76%) as compared to the usual diet, while satisfying the nutritional constraints. Additionally, the optimized diet will satisfy the micronutrient needs of Lebanese adults by significantly increasing the associated intake of vitamin A (+202%), vitamin C (+114%), calcium (+106%), iron (+77%), and folate (+174%) (Table 6).

**Table 6:** Daily values of EFPs, cost, energy, and nutrients from the daily recommended amounts of foods as per the optimized diet.

	**Unit**	**Reference**	**Optimized diet**	**Change from**
		**diet**	**quadratic**	**reference diet**
Water	L	1,370	1,280	−6%
Energy	MJ	17.5	17.5	–
GHG	kg CO_2_ eq	1.91	1.48	−22%
Cost/day	L.L.	16,700	16,700	–
Kcal	kcal	1,700	1,610	−5%
Carbs	g	191	225	+18%
Protein	g	85	103	+22%
Fat	g	78.3	52.2	−33%
Fiber	g	16.6	30	+80%
Saturated fat	g	19.4	18	−7%
Unsaturated fat	g	50	24.9	−50%
Trans fat	g	0.465	0.11	−76%
Vitamin A	mcg	916	2,770	+202%
Thiamin	mg	1.53	1.74	+14%
Riboflavin	mg	1.37	1.69	+23%
Niacin	mg	19.7	17.8	−10%
Vitamin B12	mcg	4.2	2.7	−36%
Vitamin C	mg	79.2	170	+114%
Calcium	mg	486	1,000	+106%
Iron	mg	9.18	16.3	+77%
Zinc	mg	8.54	9.84	+15%
Folate	mcg	325	890	+174%

When the aforementioned findings were discussed with the key stakeholders, it was agreed that concerted joint efforts from various entities is needed to ensure the availability, accessibility, and affordability of the highlighted food options. More specifically, three main underlying objectives were highlighted in the meeting: (1) ensuring that the recommended food options (healthy and sustainable) are heavily subsidized, (2) consolidate the support of all development agencies and partners to ensure that the prices remain affordable for families, (3) focus the incentive schemes on local production of the items constituting the bulk of this diet. To support these objectives and ultimately endorse the proper implementation of the proposed diet, key policy options, and approaches were proposed:

- Integrating the healthy and sustainable dietary scenario into a national food security plan.
- Invite the support of local and international partners.
- Subsidizing the proposed healthy and affordable dietary options using a gradual approach.
- Support local productions by incentivizing farmers to produce the recommended food options with financial incentives and potential fair bulk purchasing of crop by the government.
- Developing and implementing educational material and awareness campaigns targeting the various segments of the population and using multiple platforms with a focus on educational institutions.

## Discussion

The present paper describes the process that was used to derive a healthy, sustainable, and affordable diet for Lebanon using an optimization mathematical model to characterize an optimal diet solution that is sustainable, of low cost, and EFPs while satisfying the adequate nutrient needs for adults in Lebanon. The dimensions of sustainability that were considered in this project were the nutritional, EFPs, and cost. A consumption pattern in line with these recommendations reduces the risk of major chronic diseases, supplies adequate amounts of energy and nutrients, and reduces the environmental impact compared with the mean usual national consumption. A shift from the usual food consumption pattern to a food pattern that resembles the suggested healthy and sustainable diet is regarded as an integral component of food security and as key to achieving Sustainable Development Goals, which call for responsible production and consumption, to ensure food security and nutrition within sustainable food systems. This sustainability aspect is being recommended to be included as a 5th dimension of food security (34–36). The recommended changes in food cosumption patterns for Lebanon will result in a higher consumption of whole grain bread, bulgur, fruits, vegetables, dairy products, fish, eggs, nuts, and legumes. According to the 2015 Dietary Guidelines Advisory Committee (DGAC), a dietary pattern that is higher in plant-based foods, such as vegetables, fruits, whole grains, legumes, nuts, and seeds, and lower in animal-based foods is more health promoting and is associated with lesser environmental impact (GHG and energy, land, and water use) (37).

Although, strong evidence highlights the need to align health and environmental objectives in dietary guidelines and recommendations, only four countries; Germany, Brazil, Sweden, and Qatar, have so far included environmental sustainability aspects in their dietary guidelines (32). The healthy and sustainable diet, according to our model, may lead to a 22% cut in kgCO2eq and 6% reduction in water consumption compared to the usual national diet. This is in line with several studies that have suggested that vegan and vegetarian diets have the lowest GHG emissions, with up to 53% reduction compared to reference scenarios (38). Our results also suggest that the healthy and sustainable diet is not more expensive than the usual national diet, therefore fully affordable for the population under study. This confirms other findings that a healthier and more eco-friendly diet is not necessarily more expensive (19, 39).

From the perspective of NCDs, there are particular advantages of the healthy and sustainable optimized dietary pattern as compared to the usual national consumption. These benefits include: the decrease in the intake of trans fat (40–42) and saturated fat (43). Also, given the high fiber intake as indicated by the increase in 80% intake of fiber in the optimized diet, there would probably be additional health benefits particularly a reduction in the risk of developing coronary heart disease, stroke, hypertension, diabetes, obesity, and certain gastrointestinal disorders (44–49). These type of health benefits have been suggested by others who have recently modeled environmentally sustainable dietary scenarios, including estimating the deaths delayed or averted per year (50). The suggested healthy and sustainable dietary pattern may also contribute toward alleviating the burden of micronutrient deficiencies that are highly prevalent in Lebanon (51). The results of this study would allow for evidence based development of SFBDGs for Lebanon.

Given these results, one can reasonably ask how we might motivate sustainable dietary patterns among Lebanese adults. The EAT-Lancet report (52) highlights the need for an integrated food systems approach to reach the sustainable development goals, taking into account all steps in agricultural and aquatic production, trade, manufacturing, retailing in addition to consumption. This will involve many sectors and should have a focus of long-term achievements and development of economically, politically, technically and environmentally robust systems, taking into consideration consumer preferences. Policy-makers know that consumer behavior change would be central to any policy process aiming at integrating nutrition and sustainability (53). As such the findings of this study may help policymakers understand potential benefits of tradeoffs for promoting such diets and make investment choices while taking action to address any potential negative consequence. The findings of this study are not to facilitate “policy drift” to focus on lifestyle choices, but rather to provide a framework to support cross-sectoral food and health policy discussions, particularly in relation to dietary guidelines in Lebanon. An important element to be considered in policy recommendations related to food consumption is the taste costs of the shifts in dietary intakes. The latter has been defined as the ‘utility loss induced by a dietary change that brings a new balance between long-term health goals and short-term pleasure and hedonic rewards’ (54). Therefore, investigating the willingness and readiness of the Lebanese population to adapt the proposed shifts toward healthy and sustainable diets is an integral step toward the successful development and implementation of any policy recommendations.

## Conclusion

Shifting national food consumption patterns to promote human health, and at the same time controlling cost and mitigating environmental impact, represents an obvious “win–win” future scenario. The optimized diet suggested in this study highlights the need to increase the intakes of; whole grain bread, dark green vegetables, dairy products, and legumes, and lower intakes of; refined grain bread, meat, poultry, added sugars, saturated fat, as compared to usual national mean consumption. This diet will result in a decrease in the associated EFPs, keeping the cost constant and satisfying all necessary nutritional needs for adults. Findings highlight the need for the development of sustainable food based dietary guidelines for Lebanon to promote diets that are healthy, sustainable, culturally acceptable, and affordable and that can alleviate food insecurity among the general population. It further pushes the agenda of the Water-Energy-Food-Health Nexus forward as it proposes an opportunity for efficient resource use and reduced environmental impact all along the food supply chain to food consumption. As such, a successful transition to healthy and more sustainable dietary patterns would require concerted actions by many key stakeholders, and a diverse set of policy actions to encourage the adoption of the recommended changes across the food system. Such policy recommendations included integrating the healthy and sustainable dietary shifts into a national food security plan using a food system approach and subsidizing the proposed healthy and affordable dietary options. A few limitations ought to be considered in the interpretation of the findings of this study. The data used for usual intake dated back to 2009. However, these data come from the most recent national food consumption survey, given that, since that date, no such national surveys have been conducted. More recent studies on population subgroups in Lebanon showed that dietary intake seemed to continue to shift toward a more Western type of diet with a concomitant erosion of the traditional Lebanese diet (55–57). There are other limitations to the present study, which partially relate to the general limitations associated with using 24 h recalls to measure dietary consumption. Twenty-four hour recalls may be associated with measurement error due to inaccuracies in estimating frequencies and determination of pre-quantified food items and food portion sizes (58). Furthermore, the use of Nutritionist pro software to calculate estimations of food and nutrient intake may pose some limitations, since many factors render databases limited in terms of local applicability. Thus, it should be recognized that some degree of bias on a survey's outcome related to food variations among countries could possibly occur (59). It is also important to note that, the estimation of the EFPs required the reliance on LCAs conducted in other countries because of the absence of data in Lebanon. However, every effort was exerted to identify LCAs within neighboring countries in the MENA region, or, otherwise, use LCAs from other countries that have comparable climate and environmental conditions to Lebanon. The findings of these studies together with the increasingly dwindling environmental resources in the country further underscore the importance of the findings of this study. That said, the regular conduct of a national food-consumption survey is warranted to provide updated information on various aspects of dietary intake in Lebanon.

## Data Availability Statement

The original contributions presented in the study are included in the article/Supplementary Material, further inquiries can be directed to the corresponding author/s.

## Author Contributions

NH, FN, LN, and LJ led the conceptualization of the study. NH, RH, and SK supervised data entry, optimization, and data analysis. SK provided technical help in the use of the optimization software. RH conducted the optimization and tabulation of the data. NH and RH prepared the first draft of the manuscript. NH, FH, FN, LN, LJ, and SK critically reviewed the manuscript and contributed to the final draft of the manuscript. All authors have read and approved the final version of the manuscript.

## Conflict of Interest

The authors declare that the research was conducted in the absence of any commercial or financial relationships that could be construed as a potential conflict of interest.
