# Supplementary material for: Promoting Sustainable and Healthy Diets to Mitigate Food Insecurity Amidst Economic and Health Crises in Lebanon
Source: Front Nutr. 2021 Jun 25;8:697225. doi: 10.3389/fnut.2021.697225 (PMC8270169; doi:10.3389/fnut.2021.697225)
Supplement: Supplementary file 1 [file Table_1.DOCX]

Supplementary Material

# Supplementary Table 1

| **Cereals and grains** |  |
| --- | --- |
| Bread | Pita bread white, whole wheat pita bread, baguette, tortilla flour, rolls, pain de mie, flour, … |
| Cooked bulgur |  |
| Cooked rice | White rice, brown rice, rice cake |
| Ready to eat breakfast cereals | Bran flakes cereal, corn flakes cereal, oatmeal cereal |
| Cooked pasta | Pasta, noodles |
| **All fruits** |  |
| Fresh fruits | Apples, apricot, bananas, cherries, figs, grapes, kiwi, orange, mangos, melon, … |
| Dried fruits | Date, dried figs, raisins |
| Fresh fruit juices | 100% fresh fruit juices (orange, apple, …) |
| **All vegetables** |  |
| Dark green vegetables | Celery, lettuce, mint, parsley, spinach, swiss chard |
| Red/orange vegetables | Carrot, tomato |
| Other vegetables | Artichoke, asparagus, bell pepper, cabbage, cucumber, eggplant, onion, garlic, mushrooms, okra, peas, squash |
| **All starchy vegetables** |  |
| Potatoes | Potato, sweet potato, French fries |
| Corn | Corn kernels, cornstarch, corn tortilla, popcorn, corn flour |
| **Dairy products** |  |
| Milk | Milk (non-fat, low fat, full fat), soy milk, goat milk, milk powder |
| Yogurt |  |
| Cheese | Cheddar, feta, akkawi, brie, cottage, gruyere, mozzarella |
| Labneh |  |
| **Protein rich foods** |  |
| Meat | Beef, lamb, veal, organ meat (liver), hamburger patty |
| Poultry | Chicken (all parts) |
| Fish | Crab, tuna, white perch, grouper |
| Eggs |  |
| Legumes | Beans, chickpeas, lentils, lupins |
| Nuts & seeds | Pumpkin seeds, sunflower seeds, sesame |
| **Processed meat** | Ham, frankfurter, pastrami, sausage, pepperoni, salami, turkey |
| **Sweets** | Cakes, cookies, candies, pastries, Arabic sweets, ice cream, chocolate bars, chocolate spread |
| **Salty snacks** | Crackers, potato chips, tortilla chips |
| **Added sugar** | Honey, jam, sugar |
| **SSB excluding fresh fruits** | Bottled fruit juices, canned fruit juices, lemonade, fruits canned in heavy syrup, sodas |
| **Soda diet** |  |
| **Added fat (unsaturated)** | Vegetable oils, olive oil, olives, salad dressings, mayonnaise, tahini |
| **Saturated oil** | Butter, ghee |

*processed meat, sweets, salty snacks, SSB, and soda diet were excluded from the optimization
